# The effect of high standard uptake value in lung cancer metastatic lesions on survival

**DOI:** 10.3325/cmj.2023.64.179

**Published:** 2023-06

**Authors:** Suphi̇ Aydin, Aydın Balcı, Ahmet Dumanlı, Leyla Nesrin Acar, Murat Ayşin, Adem Gencer, Gürhan Öz, Hacer Demir, Sena Ece Davarcı

**Affiliations:** 1 Department of Thoracic Surgery, Afyonkarahisar Health Sciences University, Afyonkarahisar, Turkey; 2 Department of Pulmonology, Afyonkarahisar Health Sciences University, Afyonkarahisar, Turkey; 3 Department of Thoracic Surgery, SBÜ Ankara Keçiören Atatürk Sanatoryum Pulmonology and Thoracic Surgery Training and Research Hospital, Ankara, Turkey; 4 Department of Public Health, Katip Celebi University, Faculty of Medicine, Izmir, Turkey; 5 Department of Thoracic Surgery, Afyonkarahisar Public Hospital, Afyonkarahisar, Turkey; 6 Department of Internal Medicine and Medical Oncology, Afyonkarahisar Health Sciences University, Afyonkarahisar, Turkey

## Abstract

**Aim:**

To assess how metastatic lesions with a higher maximum standard uptake value than the primary tumor affect survival in patients with lung cancer.

**Methods:**

The study enrolled 590 stage-IV lung cancer patients treated at Afyonkarahisar Health Sciences University Hospital between January 2013 and January 2020. We retrospectively collected data on histopathological diagnosis, tumor size, metastasis site, and maximum standard involvement values of primary metastatic lesions. Lung cancers with the maximum standard uptake value of the primary tumor higher than that of the metastatic lesion were compared with lung cancers with the maximum standard uptake value of the primary tumor lower than that of the metastatic lesion.

**Results:**

In 87 (14.7%) patients, the maximum standard uptake value was higher in the metastatic lesion than in the primary lesion. These patients experienced significantly higher mortality risk in both univariate and multivariate survival analyses (adjusted hazard ratio 2.25 [1.77-2.86], <0.001) and had shorter median survival (5.0 [4.2-5.8] vs 11.0 [10.2-11.8] months, *P* < 0.001).

**Conclusions:**

The maximum standard uptake value could be a potential new prognostic factor for survival in lung cancer.

Lung cancer is the leading cause of cancer-related deaths worldwide (1). Approximately 80%-85% of lung cancers are adenocarcinomas, squamous cell carcinomas (SCC), large cell carcinomas, and unclassified carcinoma types, including non-small cell lung cancers (NSCLC) (2). TNM staging, which is based on the evaluation of tumor (T), nodal involvement (N), and metastasis (M), is the most valuable prognostic factor in the prediction of survival of both surgical and non-surgical patients with NSCLC. An important tool in performing TNM staging is positron emission tomography/computed tomography (PET/CT) (3). PET/CT is used to reliably determine primary and metastatic malignant tumors, their morphology and metabolic activity, stage, as well as recurrence and treatment response (4,5). The most widely used type of PET/CT is 18F-deoxyglucose (18F-FDG) PET/CT, which assesses the amount of FDG uptake by cells, a measure directly linked to glucose metabolism. FDG uptake in cancer cells is greater than in healthy cells. Therefore, cancer cells can be identified with 18F-FDG and visualized with PET/CT (4,6). The standard uptake value (SUV) is a semiquantitative marker of normalized radioactivity concentration, and the maximum SUV (SUVmax) is the most widely used diagnostic and prognostic parameter in clinical practice. The SUVmax on PET/CT is a marker of glucose uptake in cells. Generally, it indicates proliferative activity, metastatic potential, and the aggressiveness of tumor cells (7,8). SUVmax is strongly related to the histopathological type, tumor size (9), and survival (10). A recent systematic review has shown a negative correlation between SUVmax and prognosis in NSCLC patients (11). PET/CT has 96.8% sensitivity and 77% specificity for the differentiation of benign and malignant tissues (12). Although PET/CT is the preferred diagnostic tool in the determination of tumors, it still yields false negative and false-positive results (13,14). Low SUVmax is determined in bronchoalveolar carcinoma, carcinoma metastases containing a mucinous component, renal cell carcinoma metastases, some invasive ductal carcinoma, and invasive breast carcinoma (12), carcinoid tumors, some types of adenocarcinoma, and tumors smaller than 1 cm, causing misleading results (15). Studies have so far mostly performed SUVmax measurements in primary lung cancers and metastatic lesions, but no studies have assessed the effect of mSUVmax on survival. The primary aim of this study was to compare lung cancers with a higher maximum standard uptake value obtained by PET/CT in the primary tumor than in the metastatic lesion (pSUVmax) with lung cancers with a higher maximum standard uptake value in the metastatic lesion than in the primary tumor (mSUVmax). The secondary aim was to investigate the effects of SUVmax on survival, histopathological subtype, tumor size, and the region of metastatic involvement.

## PATIENTS AND METHODS

We retrospectively reviewed the medical records of patients diagnosed with lung cancer in the Thoracic Surgery and Thoracic Diseases Clinics of Afyonkarahisar Health Sciences University Hospital between January 2013 and January 2020. A total of 680 patients were diagnosed with stage-IV lung cancer and underwent PET/CT examinations. The study included patients who had a pathological diagnosis of lung cancer, had not started treatment, were followed up regularly, underwent PET/CT examination, had stage IV lung cancer according to the 8th TNM staging classification, and died.

Patients were not included if they had typical-atypical carcinoids, lymphoma, or other organ tumors that had spread to the lungs, if they underwent chemotherapy and/or radiotherapy before PET/CT, or surgery that could affect SUVmax, or if they had an impaired glucose metabolism, an active infection, active granulomatosis, or any other disease. After the exclusion of 90 patients who did not meet the study criteria, 590 patients were included in the study. Their pathology reports, PET/CT images, and reports were reviewed.

Oncological treatment started after the diagnosis. NSCLC patients underwent pretreatment mutation analysis. Patients with a driver mutation received targeted therapy, while those without a driver mutation received standard chemotherapy regimens according to their histological subtype. SCLC patients received standard chemotherapy regimens. NCSLC and SCLC patients received palliative radiotherapy if their metastatic (pain, cord pressure, etc) or primary tumor (obstruction, pain, etc) were symptomatic. The study complied with the Helsinki Declaration, and was approved by the Ethics Committee of Afyonkarahisar Health Sciences University.

### Statistical analysis

Descriptive statistics are summarized as mean ± standard deviation for continuous variables or as numbers and percentages for categorical variables. The normality of distribution was evaluated with a Shapiro Wilk test. The differences between the groups in continuous variables were assessed with an independent samples *t* test or a Mann-Whitney U-test. The differences between categorical variables were assessed with a χ^2^ test or Fisher exact test. Survival analyses were conducted with the Kaplan-Meier method and Cox regression analysis. The level of significance was set at *P* < 0.05. The statistical analysis was performed with SPSS Statistics for Windows, version 20.0 (IBM Corp., Armonk, NY, USA).

## RESULTS

**Figure 1:**
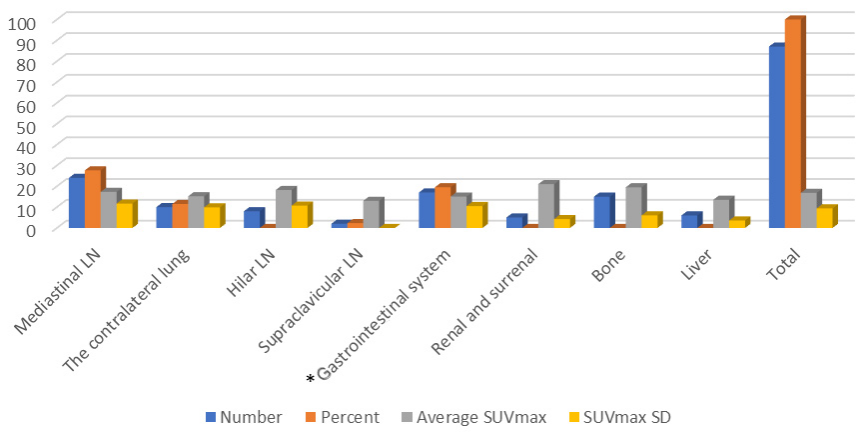
The maximum standard uptake value (SUVmax) of the metastatic lesions and metastatic regions. *Gastrointestinal system: colon, rectum, stomach, esophagus; LN – lymph node.

The final sample involved 590 patients (523 [88.6%] men). The mean age was 64.0 ± 9.5 years (range 38-88 years). All patients had widespread lymph node (LN) involvement and widespread metastases. Histopathological evaluation revealed 200 (33.9%) adenocarcinomas, 262 (44.4%) SCC, and 128 (21.7%) SCLC (Table 1). Eighty-seven (14.7%) patients had a higher SUVmax in metastatic lesions than in the primary tumor. In 264 (44.7%) patients, the size of the primary tumor was 5-7 cm (Table 2). When all 590 cases were evaluated together, pSUVmax was 15.19 ± 12.38 and the mSUVmax was 16.80 ± 9.37 (Table 1). The most frequent localization was the right lung upper lobe. This localization was present in 221 (65.5%) patients with pSUVmax and in 30 (34.5%) patients with mSUVmax (Figure 1), as well as in 63 (31.5%) patients with adenocarcinomas, 127 (48.5%) with SCC, and 31 (24.2%) with SCLC. Lesions with mSUVmax were observed most often in the mediastinal LN – in 24 (27.6%) patients, and the mean SUVmax was 17.29 ± 11.69. They were least often observed in the supraclavicular LN – in 2 (2.3%) patients, and the mean SUVmax was 13.00 ± 0. Metastasis in the renal and surrenal glands was present in 5 (5.8%) patients, and it was the region with the highest involvement, with a SUVmax of 21.00 ± 4.25 (Figure 1).

**Table 1:** Demographic characteristics of patients according to histopathological subtypes

	Total (n = 590)	Adenocarcinoma (n = 200)	SCC (n = 262)	SCLC (n = 128)
Age (years), mean ± standard deviation^+^	64.0 ± 9.5	64.3 ± 9.5	64.8 ± 9.7	61.9 ± 9.1
Sex, n (%)				
male	523 (88.6)	164 (82.0)	239 (91.2)	120 (93.7)
female	67 (11.4)	36 (18.0)	23 (8.8)	8 (6.3)

*Abbreviations: SCC – squamous cell carcinoma; SCLC – small cell lung carcinoma.

**Table 2:** Comparison of primary tumor and metastatic lesion SUVmax in terms of tumor diameter and survival periods

	Total		Adenocarcinoma		SCC		SCLC	
	pSUVmax (n = 503)^+^	mSUVmax (n = 87)^+^	pSUVmax (n = 173)	mSUV max (n = 27)	pSUVmax (n = 222)	mSUVmax (n = 40)	pSUVmax (n = 108)	mSUVmax (n = 20)
**Tumor diameter**^†^ (cm)								
**<3**	17.75 ± 6.61 (n = 54)	19.69 ± 9.36 (n = 7)	10.91 ± 6.11 (n = 19)	15.62 ± 3.96 (n = 3)	14.44 ± 5.89 (n = 24)	17.76 ± 7.36 (n = 3)	12.88 ± 10.10 (n = 11)	16.96 (n = 1)
**3-5**	15.56 ± 19.09 (n = 183)	15.43 ± 8.88 (n = 30)	17.33 ± 29.99 (n = 62)	16.31 ± 7.13 (n = 8)	14.16 ± 6.20 (n = 83)	13.38 ± 5.31 (n = 14)	15.17 ± 7.74 (n = 38)	18.03 ± 14.50 (n = 8)
**5-7**	14.90 ± 7.62 (n = 225)	16.88 ± 10.38 (n = 39)	12.70 ± 6.75 (n = 69)	19.29 ± 14.95 (n = 13)	15.98 ± 6.76 (n = 107)	16.54 ± 7.80 (n = 18)	16.03 ± 10.12 (n = 49)	13.73 ± 5.66 (n = 8)
**>7**	17.10 ± 6.04 (n = 41)	18.52 ± 7.11 (n = 11)	16.47 ± 6.00 (n = 23)	16.50 ± 3.62 (n = 3)	17.68 ± 6.32 (n = 8)	20.44 ± 9.18 (n = 5)	17.63 ± 6.20 (n = 10)	17.36 ± 7.35 (n = 3)
**Average SUVmax^†^**	15.19 ± 12.38	16.80 ± 9.37	14.68 ± 17.90	17.64 ± 10.91	15.35 ± 6.46	16.01 ± 7.27	15.82 ± 8.54	17.19 ± 11.04
**Survival** **(months)^‡^**	11.0 (10.2-11.8)	5.0 (4.2-5.8)	10.7 (9.2-12.2)	3.7 (3.4-3.9)	11.0 (9.7-12.3)	6.0 (5.3-6.7)	10.8 (8.5-13.1)	5.3 (2.4-8.2)
	*P* < 0.001		*P* < 0.001		*P* < 0.001		*P* = 0.001	

*Abbreviations: SCC – squamous cell carcinoma; SCLC – small cell lung carcinoma; PET/CT –positron emission tomography/computed tomography; SUVmax – maximum standard uptake value; pSUVmax – higher maximum standard uptake value in the primary tumor than in the metastatic lesion; mSUVmax – higher maximum standard uptake value in metastatic lesion than in the primary tumor.

†one-way ANOVA test (mean ± standard deviation)

‡Kaplan-Meier (log rank test); median (95% confidence interval).

**Figure 2:**
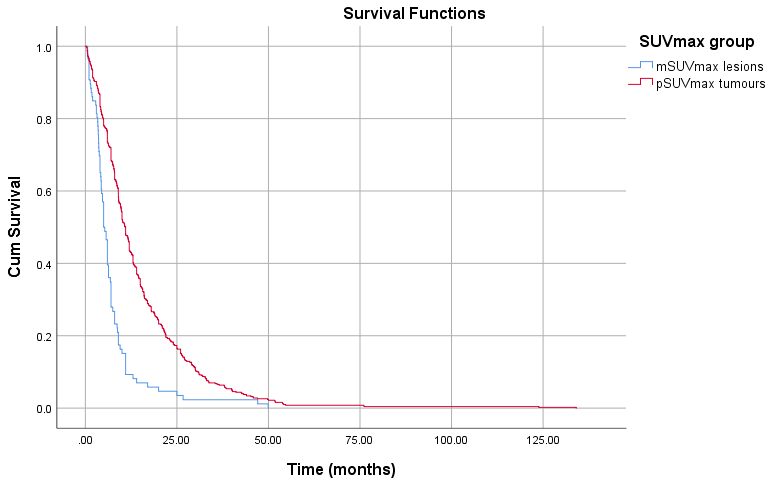
Kaplan-Meier survival curve for patients with tumors with the maximum standard uptake value of the primary tumor higher than that of the metastatic lesion (pSUVmax) and those with the maximum standard uptake value of the primary tumor lower than that of the metastatic lesion (mSUVmax)

mSUVmax and pSUVmax patient groups did not significantly differ in age, sex, histopathological subtype distribution, and lesion size (Table 3). The median survival was 5.0 (4.2-5.8) months for cancers with mSUVmax, and 11.0 (10.2-11.8) months for cancers with pSUVmax (*P* < 0.001) (Table 1). While one-year survival in patients with mSUVmax lesions was 9.3%, in patients with pSUVmax lesions it was 43.5%. We found high mSUVmax to be an important prognostic factor when considering survival time (Figure 2). When all patients were examined, no significant difference in survival was found according to all histological subtypes (*P* = 0.529). The one-year survival rates of the patient groups were also similar. The one-year survival rate of patients with adenocarcinoma was 38.7%, that of patients with SCC was 39.7%, and that of patients with SLCL was 35.9%. Univariate analysis showed no significant difference in sex, tumor diameter, or histopathological subtype. These variables were included in the multivariate Cox regression model to correct for possible effects. In multivariate Cox regression, age increased the mortality risk (hazard ratio 1.02, 95% confidence interval 1.01-1.02)). In lung cancer patients with mSUVmax, the mortality risk increased more than 2-fold (hazard ratio 2.25; 95% confidence interval 1.77-2.86) (Table 4).

**Table 3:** Age, sex, histological subtypes, and tumor size in patients with the maximum standard uptake value of the primary tumor higher than that of the metastatic lesion (pSUVmax) and those with the maximum standard uptake value of the primary tumor lower than that of the metastatic lesion (mSUVmax)

	mSUVmax lesions n = 87	pSUVmax tumors n = 503	p
Age (years), mean ± standard deviation	65.1 ± 9.5	63.8 ± 9.5	0.255^†^
Sex, n (%)			
male	77 (88.5)	446 (88.7)	0.965^‡^
female	10 (11.5)	57 (11.3)	
Histopathological subtypes, n (%)			
adenocarcinoma	27 (31.0)	173 (34.4)	0.826^‡^
SCC	40 (46.0)	222 (44.1)	
SCLC	20 (23.0)	108 (21.5)	
Tumor diameter in cm, n (%)			
<3	7 (8.1)	54 (10.7)	0.514^‡^
3-5	30 (34.5)	183 (36.4)	
5-7	39 (44.8)	225 (44.7)	
>7	11 (12.6)	41 (8.2)	

*Abbreviations: SCC – squamous cell carcinoma; SCLC – small cell lung carcinoma.

†t-test.

‡χ^2^ test.

**Table 4:** Univariate and Cox regression analysis of the factors affecting survival

	Univariate analysis (HR [95% CI])	p	Multivariate analysis multivariate (adjusted HR [95% CI])	p
Male sex	1.08 (0.84-1.39)	0.537	1.09 (0.84-1.41)	0.513
Age	1.02 (1.01-1.03)	<0.001	1.02 (1.01-1.02)	<0.001
mSUVmax > pSUVmax	2.29 (1.81-2.90)	<0.001	2.25 (1.77-2.86)	<0.001
Tumor diameter >7 cm	1.06 (0.73-1.53)	0.752	0.86 (0.59-1.25)	0.442
SCLC	1.12 (0.91-1.39)	0.265	1.21 (0.98-1.50)	0.074
Adenocarcinoma	1.04 (0.87-1.25)	0.629	1.08 (0.90-1.31)	0.384
SCC	0.95 (0.79-1.14)	0.535	0.91 (0.76-1.11)	0.198

*Abbreviations: SCC – squamous cell carcinoma; SCLC – small cell lung carcinoma; pSUVmax – higher maximum standard uptake value in the primary tumor than in the metastatic lesion; mSUVmax – higher maximum standard uptake value in the metastatic lesion than in the primary tumor, HR – hazard ratio; CI – confidence interval.

## DISCUSSION

In this study, patients who had a higher SUVmax in the metastatic lesion than in the primary lesion experienced shorter survival. Distant metastasis in lung cancer is known to be one of the strongest prognostic factors affecting survival. As the treatment strategy directly depends on the tumor stage, correct staging of distant metastases is important (16). Although lung cancer generally has a similar incidence in men and women, female sex is an independent positive prognostic factor affecting survival in lung cancer (17). SCLC and SCC are more common in men, and adenocarcinoma is more common in women. The incidence differs between the sexes, which may be explained by hormonal and genetic factors (18,19). In the current study, although lung cancer was more often found in men, no significant difference in survival was found between the sexes. SCLC and SCC were most often diagnosed in men, and adenocarcinoma in women. Differential activation of oncogenes and inactivation of tumor suppressor genes were higher in primary tumors than in metastases, with higher amplification levels in metastases (20). The general age of diagnosis of lung cancer is around 70 years (21,22). The mean age of the patients was 64.0 ± 9.5 years (range, 38-88 years), which is consistent with the literature findings. With each unit increase in age, the risk of mortality increased by 2%. In the current study, the primary tumor SUVmax was 14.68 ± 17.90 in adenocarcinoma, 15.35 ± 6.46 in SCC, and 15.82 ± 8.54 in SCLC, which is similar to the literature findings (23). Lesions with mSUVmax were found in the gastrointestinal system and bone tissue, and the distant metastasis region with the highest SUVmax was the bone tissue. The most frequent sites of metastasis reported in the study by Riihimaki et al (24) were the bone (39%) and the respiratory system (22%) in patients with adenocarcinoma, and the nervous system (47%) and liver (35%) in patients with SCLC. Patients with bone metastasis had shorter survival. In a study that included 17 431 patients, survival in non-metastatic lung cancer was 12 months in men and 14 months in women, and in metastatic lung cancer, it was 4 months in men and 5 months in women (24). In the current study, the overall median survival was 11 months in lung cancers with pSUVmax and 5 months in lung cancers with mSUVmax. The differences in survival between the current and previous studies can be attributed to the histological subtypes of lung cancer included in the studies, patient sex ratios, and the treatment methods applied.

A limitation of this study is the accuracy of PET/CT for distant metastasis staging in lung cancers, which remains a matter of debate. Differences in reporting may have been caused by several reasons (technical parameters, clinicians making the interpretations, criteria defining positive PET/CT results), preventing the standard interpretation of the PET/CT results by a single team.

Evaluation of long-term survival outcomes in cancer patients is important for treatment planning and cancer control. The results of this study suggest a relationship between mSUVmax and survival, possibly due to tumor cell genetics and the turnover of tumor cells. SUVmax is a potential new prognostic factor for survival in lung cancer; however, studies with a larger number of patients are needed to confirm our results.
